# Antenatal care service delivery and factors affecting effective tetanus vaccine coverage in low- and middle-income countries: Results of the Maternal Immunisation and Antenatal Care Situational analysis (MIACSA) project

**DOI:** 10.1016/j.vaccine.2020.05.025

**Published:** 2020-07-14

**Authors:** M.L. Giles, E. Mason, F.M. Muñoz, A.C. Moran, P. Lambach, S. Merten, T. Diaz, M. Baye, M. Mathai, J. Pathirana, S. Rendell, Ö. Tunçalp, J. Hombach, N. Roos

**Affiliations:** a Department of Obstetrics and Gynaecology, Monash University, Melbourne, Australia; b London School of Hygiene and Tropical Medicine, London, UK; c Section Infectious Diseases, Molecular Virology and Microbiology, Baylor College of Medicine, Houston, TX, USA; d Epidemiology, Monitoring and Evaluation (EME), Department of Maternal, Newborn, Child and Adolescent Health and Ageing, World Health Organization, Geneva, Switzerland; e Department of Immunization, Vaccines and Biologicals (IVB), World Health Organization, Geneva, Switzerland; f Swiss Tropical and Public Health Institute and University of Basel, Basel, Switzerland; g Coordinator of the National Program to Combat Maternal, Newborn and Child Mortality, Ministry of Public Health, Cameroon; h Department of International Public Health, Liverpool School of Tropical Medicine, Liverpool, United Kingdom; i Medical Research Council: Respiratory and Meningeal Pathogens Research Unit and Department of Science and Technology/National Research Foundation: Vaccine Preventable Diseases, Faculty of Health Sciences, University of the Witwatersrand, Johannesburg, South Africa; j Department of Anthropology, University of Pennsylvania, Philadelphia, USA; k Department of Sexual and Reproductive Health and Research Including UNDP/UNFPA/UNICEF/WHO/World Bank Special Programme of Research, Development and Research Training in Human Reproduction, WHO, Geneva, Switzerland; l Karolinska Institutet, Department of Medicine, Clinical Epidemiology Division, Stockholm, Sweden

**Keywords:** Pregnancy, Antenatal care, Tetanus, Vaccination, MIACSA

## Abstract

**Objectives:**

To map the integration of existing maternal tetanus immunization programmes within antenatal care (ANC) services for pregnant women in low- and middle-income countries (LMICs) and to identify and understand the challenges, barriers and facilitators associated with high performance maternal vaccine service delivery.

**Design:**

A mixed methods, cross sectional study with four data collection phases including a desk review, online survey, telephone and face-to-face interviews and in country visits was undertaken between 2016 and 2018. Associations of different service delivery process components with protection at birth (PAB) and with country groups were established. PAB was defined as the proportion of neonates protected at birth against neonatal tetanus. Regression analysis and structural equation modelling was used to assess associations of different variables with maternal tetanus immunization coverage. Latent class analysis (LCA), was used to group country performance for maternal immunization, and to address the problem of multicollinearity.

**Setting:**

LMICs.

**Results:**

The majority of LMICs had a policy on recommended number of ANC visits, however most were yet to implement the WHO guidelines recommending eight ANC contacts. Countries that recommended > 4 ANC contacts were more likely to have high PAB > 90%. Passive disease surveillance was the most common form of disease surveillance performed but the maternal and neonatal morbidity and mortality indicators recorded differed between countries. The presence of user fees for antenatal care and maternal immunization was significantly associated with lower PAB (<90%).

**Conclusions:**

Recommendations include implementing the current WHO ANC guideline to facilitate increased opportunities for vaccination during each pregnancy. Improved utilisation of ANC services by increasing the demand side by increasing the quality of services, reducing any associated costs and supporting user fee exemptions, or the supply side can also enhance utilisation of ANC services which are positioned as an ideal platform for delivery of maternal vaccines.

## 1 Background

In 2015, an estimated 303,000 women died from pregnancy-related causes [1], and 2.6 million babies were stillborn [2]. In 2017 an estimated 2.5 million babies died in the first month of life [3]. Many of these deaths would be preventable through increased access to and use of quality health care during pregnancy and childbirth. Building on the progress made by the Millennium Development Goals (MDGs) in 2015, the Sustainable Development Goals (SDG) were launched to guide the eradication of poverty, hunger, illiteracy, and disease [4]. The third SDG on good health and wellbeing, aims to end preventable deaths of newborns and children under five years of age by 2030, with a target to further reduce neonatal mortality to at least as low as 12 per 1,000 live births and under five mortality to at least as low as 25 per 1,000 live births [5]. Infectious diseases, particularly pneumonia and sepsis are leading causes of death in children under five years of age [6], some of which may be preventable by maternal immunization.

Deaths during the neonatal period constitute almost 50% of the total deaths occurring in children under five years, with little progress made in the past decades [7]. The Maternal and Neonatal Tetanus Elimination (MNTE) initiative, launched in 1989, was the first maternal immunization programme to be recommended globally [8]. Along with safe birthing practices this has contributed to over 700,000 lives saved and as of today only 13 countries have yet to eliminate maternal and neonatal tetanus [8]. Other vaccines being recommended during pregnancy include influenza and in many high-income settings pertussis [9], [10], [11], [12], [13], [14], [15]. For vaccination to be an effective intervention strategy during pregnancy, it relies on access to health care during pregnancy, maternity care providers recommending the intervention, women understanding and accepting the intervention, and vaccination of the pregnant woman.

Antenatal care (ANC) is accepted as the natural entry point for interventions during pregnancy, such as maternal immunization, and provides important opportunities for the prevention, identification and treatment of diseases affecting pregnant women and their babies. Despite progress made in ANC utilization, UNICEF estimates that in 2010–2016, only 61.8% of women globally received at least four antenatal care visits [16]. This figure is even lower in regions with the highest rates of maternal and perinatal mortality, such as sub-Saharan Africa (52%) and South Asia (46%) [16]. Not only is the number of contacts during pregnancy important but also the timing of the first ANC visit. Ensuring the first ANC visit is in the first trimester, is essential to optimising health outcomes for women and children. Global estimates between 1990 and 2013 report early ANC visit as 24% in low income countries compared to 82% in high income countries [17]. In 2016, the World Health Organization (WHO) issued new recommendations to improve ANC and to reduce the risk of stillbirths and pregnancy complications [18]. Central to these new recommendations is the focus on a minimum of eight contacts during pregnancy and one ultrasound scan before 24 weeks of gestation, ensuring a healthy pregnancy for mother and baby leading to a positive birth and motherhood experience [18].

In addition to tetanus and influenza, there are new maternal vaccine candidates in development such as against respiratory syncytial virus (RSV) and group B streptococcus (GBS). In low- and middle-income countries RSV and GBS account for a significant burden of disease [19], [20]. RSV is the most important cause of viral lower respiratory tract disease in infants globally. In a systematic review, it was estimated that in 2015 RSV infection was responsible for 33.1 million episodes of RSV acute lower respiratory infection, resulting in 1.4 million hospital admissions in children less than six months of age and 27,300 in-hospital deaths, a significant proportion of these in the first month of life [19]. GBS is an important cause of neonatal sepsis and meningitis, particularly in the first 3 months of life. In 2015, there were an estimated 90,000 deaths in infants less than three months of age, 57,000 fetal infections and/or stillbirths (defined as a dead borne fetus weighing > 1000gm and/or > 28 weeks gestational age and/or body length of 35 cm) and 33,000 cases of invasive GBS disease in pregnant or post-partum women (including endometritis, chorioamnionitis and sepsis) [20].

If these vaccine candidates prove to be safe and efficacious they may be recommended for pregnant women in the future. In anticipation of this, there is an urgent need to describe the current landscape of antenatal care service provision and better understand the optimal ways to deliver vaccines, including the value of using antenatal care services as a delivery platform.

The WHO, supported by the Bill & Melinda Gates Foundation, commenced work in mapping strengths of existing maternal tetanus immunization programmes through the Maternal Immunization and Antenatal Care Situational Analysis (MIACSA) project. This project aims to identify and understand the challenges from, barriers to and facilitators of successful maternal immunization in low- and middle-income countries (LMICs) [20]. This project also sets out to develop a typology of existing health systems in terms of vaccine delivery strategies to pregnant women and the attributes associated with high performance maternal vaccine service delivery in LMICs. An important goal is to identify what aspects of antenatal care need to be strengthened and what gaps need to be addressed to inform the introduction of additional maternal vaccines particularly into antenatal care services.

## 2 Methods

### 2.1 Study design and data collection

A detailed overview of the project methodology has been published previously [21]. In summary, between November 2016 and September 2018, a mixed methods cross sectional study was carried out with four components. The four components did not run for the entire study period between 2016 and 2018, although there was considerable overlap in time of data collection between Phase III and IV. These four components are briefly summarised below.

(1) Phase I - A desktop review of pre-defined maternal and child health indicators and World Bank Data for economic level in 137 low- and middle income countries (LMICs)

The following databases were used to extract data; Demographic and Health Surveys (DHS)/Multiple Indicator Cluster Surveys (MICS), WHO/United Nations Children’s Fund (UNICEF) estimates of national immunization coverage, WHO/UNICEF Joint Reporting Forms (JRF), Maternal Neonatal Tetanus Elimination (MNTE) database, WHO Maternal, Newborn, Child, and Adolescent Health (MNCAH) policy surveys, GAVI web page for eligibility, United Nations (UN) Inter-agency Group for Child Mortality Estimation, trends in maternal mortality: 1990 to 2015 and United Nations, Department of Economic and Social Affairs, Population Division. The number of countries in the databases and time periods reviewed varies. The information used in this analysis is based on the most recent surveys at the time of the analysis.

(2) Phase II - Global online survey sent to 116 LMICs

An 18-item online survey was developed collecting data on service delivery models of maternal tetanus vaccination, programme funding, disease surveillance, vaccine safety surveillance and maternal vaccines other than tetanus. The survey was not sent to the WHO European region which decided not to participate in this project because the MNTE initiative is not a priority there.

(3) Phase III - Telephone and face to face interviews

A 91-item survey was developed by the MIACSA Expert Advisory Panel. The telephone interview was pilot tested in two countries (Sri Lanka and Tanzania), and thereafter adapted based on participant comments and administered via in-depth telephone and face-to-face interviews with Expanded Programme on Immunisation (EPI) and Maternal, Newborn and Child health (MNCH) programme officers in a sample of countries. Data from the pilots was included in the final analysis. Of the 26 interviews, 21 were completed entirely over the phone. The remaining 5 were completed partly or fully in country. These included Ethiopia, Fiji, Bhutan, Thailand and Morocco.

(4) Phase IV- In-country visits

In-country visits were conducted in ten selected countries. Countries were identified based on a system developed by the researchers to stratify countries into four groups according to maternal tetanus vaccination performance measured as protection at birth (PAB) and antenatal care performance (measured as ANC4+ coverage). A cut-off of PAB of 90% was used to divide countries into high and low maternal tetanus vaccination performers. For antenatal care performance, high and low performance was defined as either above or below the median ANC4+ coverage. The final country selection tried to ensure representation from all WHO regions, including high-performing countries, MNTE priority countries and countries with high ANC4+ coverage. Data collected included from in depth key informant interviews, focus group discussions, and health care facility observations. In each country, between 6 and 14 health facilities, identified by the Ministry of Health, were visited.

### 2.2 Key definitions

**PAB**: is the proportion of neonates protected at birth against neonatal tetanus, by combining data on the number of tetanus vaccine doses received by the mother by the last baby born, interval between doses, and time since last dose (using card or verbal history). PAB was used as dichotomous variable: low: PAB < 90% vs. high: PAB>=90%.

**ANC performance**: The proportion of pregnant women who attended at least one ANC visit during their last pregnancy (ANC1) and the proportion of pregnant women who attended four or more ANC visits during their last pregnancy (ANC4+) were used.

**EPI performance:** Data from the desk review on Diphtheria-Pertussis-Tetanus (DPT)/third dose of pentavalent (Penta3) vaccines were used.

TT2+: Proportion of pregnant women receiving at least two doses of Tetanus toxoid containing vaccine

### 2.3 Statistical analysis

All data were imported into STATA V.15 (StataCorp LCC, Texas) for analyses. All analyses were conducted using non-missing data. Summary measures (proportions, means, medians and standard errors) were obtained for all variables of interest.

Countries were then grouped in two ways; (1) according to high and low-PAB coverage and (2) according to selected MNCH and EPI performance indicators. In order to create country groups based on a combination of MNCH and EPI performance indicators, latent class analysis (LCA), was used to enable the characterization of an unobserved (latent) variable through analysis of the structure of the relationship among several observed variables [22]. LCA allowed multiple indicators to simultaneously contribute to the definition of country groups. LCA therefore was able to address the problem of multi-collinearity. The variables included in the LCA model included PAB, TT2+, DPT3, ANC1, ANC4+, neonatal mortality rate and maternal mortality rate. The LCA generated four country groups defined as;

Group 1: Currently ***very limited potential*** to protect mothers and their young children from vaccine-preventable infections (limited ANC and EPI performance)
Group 2: ***Limited potential*** to protect mothers and their young children from vaccine-preventable infections (moderate ANC and EPI performance)
Group 3: ***Moderate potential*** to protect of mothers and their young children from vaccine-preventable infections (mostly successful ANC and EPI performance)
Group 4: ***High potential*** for protection of mothers and their young children from vaccine-preventable infections (successful ANC and EPI performance)

Fisher’s exact test was used to establish differences in service delivery process components (1) by PAB coverage (PAB < 90% vs. PAB>=90%) and (2) by country groups (Group 1 vs. others; Group 2 vs. others; Group 3 vs. others; Group 4 vs. others).

## 3 Results

**Fig. 1:**
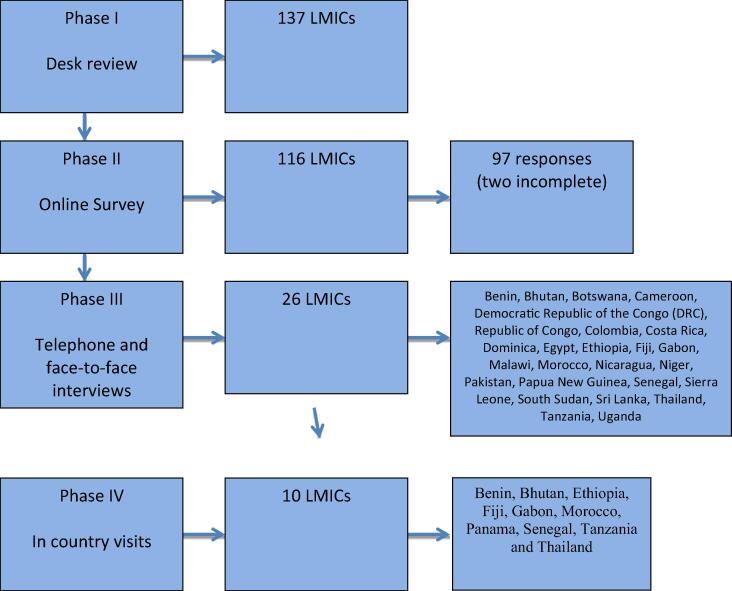
Four phases of the project and number of countries participating.

The online survey (sent to 116 LMIC countries) was answered by 97 countries, of which two provided incomplete responses. Twenty-six countries participated in the telephone survey, and week-long in-country visits took place in ten countries. A total of 96 health facility visits and interviews with health facility managers occurred during the in-country visits (Fig. 1).

### 3.1 Antenatal care policy and targets

**Fig. 2:**
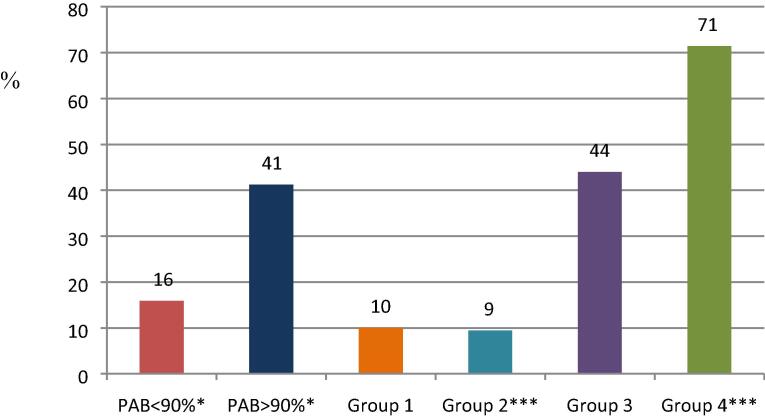
Percent of countries with a policy of >4 ANC visits. Data from online survey among 88 countries. Group 1 = very limited potential to protect mothers and young children against vaccine-preventable diseases, Group 2 = limited potential, Group 3 = moderate potential, Group 4 = high potential, PAB = protection at birth. Fisher’s Exact P-values: * <0.05, *** <0.001.

Based on Phase I (the desk review), information on the minimum number of recommended antenatal visits contained within the ANC policy was available for 122/137 (89%) countries (see Fig. 2).

Based on Phase II (the online survey), 88/95 (93%) countries provided information on minimum number of recommended ANC visits. Among these 88 countries, 15 (17.0%) recommended eight or more visits in line with the new WHO recommendation, while 15 (17.0%) countries recommended between five and seven visits. Fifty-seven countries reported having a policy of four visits (64.7%), the remaining had three visits.

Countries with a policy recommending > 3 ANC visits were more likely to have higher PAB coverage (>=90% vs < 90%, Fisher’s exact P-value = 0.020). A policy of > 4 ANC visits was also more common among group 3 (high potential) countries (Fisher’s exact P-value < 0.001) and less common among group 2 (limited potential) countries (Fisher’s exact P-value 0.001).

### 3.2 Antenatal care service delivery including number of visits, timing of visits and outreach

**Fig. 3:**
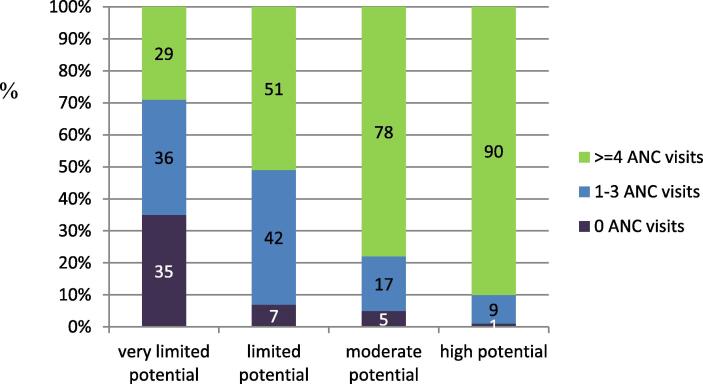
Percentage of women with 0, 1–3, or 4 or more ANC visits according to the most recent national data available, by country group (telephone survey, N = 26).

Although nearly all countries had a policy on the recommended minimum number of visits during pregnancy, few countries (12/26; 46.2%) which responded to the telephone interview were able to provide data on the proportion of women who attended a predefined number of visits including the respective timing of these visits. The number of women never accessing ANC services was considerable among countries with very limited to limited potential to protect mothers and children from vaccine preventable diseases, 35% and 7% respectively (Fig. 3).

Importantly, countries recommending > 4 antenatal visits were more likely to have high performance for maternal tetanus immunization defined as PAB > 90% (p = 0.02) and were more likely to be group 4 countries (high potential for protection of mothers and their young children from vaccine-preventable infections) (p = 0.001).

All countries provided ANC services at fixed health facilities. Government outreach programmes were offered in 55/95 (57.9%) countries, including ANC services. This was more common among group 2 (limited potential) countries (Fisher’s exact p-value 0.078) and less in group 4 (high potential, p-value 0.067), but the difference was only marginally significant (Table 1). There was no association with PAB coverage.

**Table 1:** Government outreach programmes offering ANC services. Data from online survey, 95 countries.

Outreach, ANC	PAB < 90%	PAB>=90	Group 1	Group 2	Group 3	Group 4	Total
No	18 (40)	14 (37.8)	6 (54.5)	9 (28.1)	10 (38.5)	15 (57.7)	40 (42.1)
Yes	27 (60)	23 (62.2)	5 (45.5)	23 (71.9)	16 (61.5)	11 (42.3)	55 (57.9)
Total	45 (1 0 0)	37 (1 0 0)	11 (1 0 0)	32 (1 0 0)	26 (1 0 0)	26 (1 0 0)	95 (1 0 0)
Fisher's exact P-value		1	0.518	0.078	0.816	0.067	

More detailed information about the provision of outreach services was available from the telephone interviews with 26 countries. Among the countries participating in the telephone interviews, six countries provided mobile or outreach services specifically for ANC; in eight countries ANC was included in other outreach activities, and seven countries provided both. During country visits, wide variation was observed in the content and frequency of outreach ANC services between countries and within a given country. Additionally, the majority of ANC outreach in countries visited provided a limited package of ANC services, including some or all of the following elements: blood pressure measurement, iron and folic acid supplementation, malaria prophylaxis (where applicable), counselling, prevention of mother-to-child transmission of HIV and referral of women to the nearest health facility for laboratory testing.

### 3.3 ANC service package and quality of antenatal care

According to the telephone interviews 14/22 (63.6%) countries provided at least 11 different ANC interventions in their ANC service package. The list of ANC interventions asked about included counselling on diet, iron and folic acid supplementation, vitamin A supplementation, screening for; anemia, urinary tract infection/bacteriuria, intimate partner violence, gestational diabetes, tobacco use, substance abuse, HIV, syphilis and tuberculosis, symphysis fundal measurement, ultrasound scan for gestational age estimation and screening for birth defects, antibiotic prophylaxis for prevention of recurrent urinary tract infections, screening for blood type and Rh, administration of anti-D, preventive anthelminthic treatment, intermittent preventive treatment during pregnancy (IPTp) for malaria, pre-exposure prophylaxis for HIV prevention (PrEP), counselling on danger signs in pregnancy, and birth planning. Four countries didn’t respond to the question. The three most common services offered by more than 90% of the health facilities visited, were iron and folic acid supplementation, dietary counselling, and screening for HIV. The country visits revealed a higher number of interventions offered in the ANC package in high potential countries (group 4 countries mentioned on average 12.5 essential interventions compared to 9 in the other countries, *P*–value from Wilcoxon rank sum test = 0.010, telephone interviews).

The existence and functioning of the ANC referral systems was explored in both the telephone interviews with 26 countries and the visits to 96 facilities during the country visits. National level programme managers from 25/26 countries that provided information through telephone interviews reported having a functional ANC referral system in place for high risk or complicated deliveries. Of the 96 facilities visited, 92 (95.8%) reported having a system in place for referring pregnant women to a higher level of care. The four facilities that did not report referring women were themselves reference hospitals and, thus, did not initiate referrals.

### 3.4 Funding sources and ANC coverage and quality

**Fig. 4:**
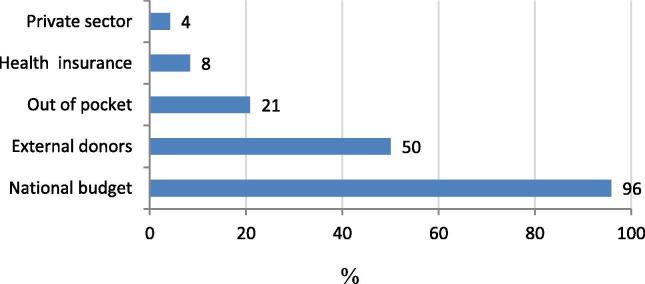
Percentage of countries reporting different ANC funding sources, information from telephone interviews (24 countries).

Most countries responding to the telephone interviews (23/24; 95.8%) reported that funding for ANC was dependent primarily upon the national budget, while 12/24 (50%) countries also benefitted from external donor funding for ANC (Fig. 4). Overall, one in five countries indicated that ANC services were partly funded by out-of-pocket payments.

The online survey in 95 countries provided data on user-fee exemptions for ANC and for maternal tetanus immunization. In 72/95 countries (75.8%), pregnant women were exempt from ANC user fees, and in all but one country, maternal tetanus immunization was free. In 12/95 (12.6%) countries, user fee exemptions for ANC were in place only for some groups in the population and in 11/95 (11.6%) countries, no user-fee exemptions existed. The imposition of user fees for antenatal care services was significantly associated with lower PAB coverage (Fisher’s exact p-value 0.037).

### 3.5 Disease surveillance

**Fig. 5:**
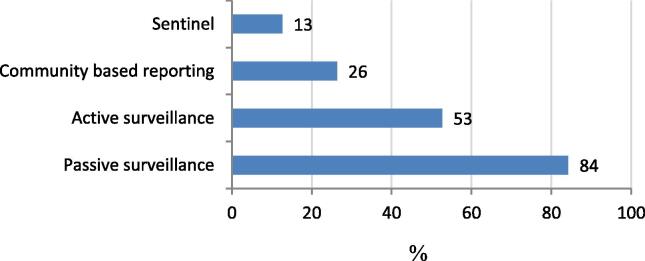
Percent of countries performing different types of disease surveillance for maternal and neonatal tetanus, data from online survey in 95 countries.

Passive surveillance was the most common form of disease surveillance performed (Fig. 5). The morbidity and mortality indicators, which were recorded, differed between countries (Table 2). According to the online survey, most common were surveillance for neonatal tetanus (91/95; 96%) and maternal deaths (84/95; 88%). Maternal and neonatal deaths were less often recorded in group 1 countries (Fisher’s exact p-value 0.003 and 0.045, respectively). Surveillance for congenital rubella, neonatal deaths and neonatal sepsis was less common overall, but more common in group 4 countries (high potential to protect mothers and their young children from vaccine-preventable infections). In terms of private facilities reporting cases of tetanus, 16/26 (62%) countries confirmed that they report tetanus cases.

**Table 2:** Disease surveillance (online survey, 95 countries).

*a - Neonatal tetanus*							
	PAB < 90%	PAB>=90	Group 1	Group 2	Group 3	Group 4	Total
No	2 (4.4)	0 (0)	1 (9.1)	0 (0)	1 (3.8)	2 (7.7)	4 (4.2)
Yes	43 (95.6)	37 (1 0 0)	10 (90.9)	32 (1 0 0)	25 (96.2)	24 (92.3)	91 (95.8)
	45 (1 0 0)	37 (1 0 0)	11 (1 0 0)	32 (1 0 0)	26 (1 0 0)	26 (1 0 0)	95 (1 0 0)
Fisher's exact		0.499	0.394	0.297	1.000	0.301	

*b -Congenital rubella syndrome*							
No	22 (48.9)	9 (24.3)	7 (63.6)	14 (43.8)	8 (30.8)	4 (15.4)	33 (34.7)
Yes	23 (51.1)	28 (75.7)	4 (36.4)	18 (56.3)	18 (69.2)	22 (84.6)	62 (65.3)
	45 (1 0 0)	37 (1 0 0)	11 (1 0 0)	32 (1 0 0)	26 (1 0 0)	26 (1 0 0)	95 (1 0 0)
Fisher's exact		**0.039**	**0.045**	0.254	0.809	**0.016**	

*c -Neonatal sepsis*							
No	35 (77.8)	25 (67.6)	9 (81.8)	27 (84.4)	16 (61.5)	13 (50)	65 (68.4)
Yes	10 (22.2)	12 (32.4)	2 (18.2)	5 (15.6)	10 (38.5)	13 (50)	30 (31.6)
	45 (1 0 0)	37 (1 0 0)	11 (1 0 0)	32 (1 0 0)	26 (1 0 0)	26 (1 0 0)	95 (1 0 0)
Fisher's exact		0.327	0.493	**0.020**	0.459	**0.026**	

*d. -Neonatal deaths*							
No	16 (35.6)	4 (10.8)	5 (45.5)	9 (28.1)	5 (19.2)	1 (3.8)	20 (21.1)
Yes	29 (64.4)	33 (89.2)	6 (54.5)	23 (71.9)	21 (80.8)	25 (96.2)	75 (78.9)
	45 (1 0 0)	37 (1 0 0)	11 (1 0 0)	32 (1 0 0)	26 (1 0 0)	26 (1 0 0)	95 (1 0 0)
Fisher's exact		**0.011**	**0.050**	0.288	1.000	**0.011**	

*e -Maternal deaths*							
No	8 (17.8)	3 (8.1)	5 (45.5)	3 (9.4)	2 (7.7)	1 (3.8)	11 (11.6)
Yes	37 (82.2)	34 (91.9)	6 (54.5)	29 (90.6)	24 (92.3)	25 (96.2)	84 (88.4)
	45 (1 0 0)	37 (1 0 0)	11 (1 0 0)	32 (1 0 0)	26 (1 0 0)	26 (1 0 0)	95 (1 0 0)
Fisher's exact		0.330	**0.003**	0.745	0.722	0.279	

## 4 Discussion

The results from the MIACSA project in LMICs demonstrate that for maternal tetanus immunization, the majority of countries despite having a policy on recommended number of ANC visits, had yet to implement the WHO guidelines recommending eight antenatal contacts [18]. Importantly, countries recommending > 4 antenatal visits were more likely to have high performance for maternal tetanus immunization defined as PAB > 90% and were more likely to be group 4 countries (high potential for protection of mothers and their young children from vaccine-preventable infections).

If future maternal vaccines are to be delivered to pregnant women via the ANC platform then ANC service delivery components including the number of contacts and timing of contacts are important. Some maternal vaccines may require administration during particular gestational windows, such as RSV vaccine (clinicalTrials.gov NCT02624947) so a system’s capacity to record timing of ANC visits is important for identifying gaps and targeting campaigns to maximise attendance at recommended time frames to optimise implementation of maternal immunization during ANC. The WHO antenatal care guidelines concentrate most of the eight recommended contacts in the third trimester. Indicators about the frequency of visits are frequently collected, but the findings from the MIACSA project highlight that information about the timing of ANC visits by trimester is rarely available and needs to be included in future monitoring efforts, should new maternal vaccines require a specific time window for administration. Given the potential importance of accurately estimating gestational age, and potentially narrow gestational windows for future maternal vaccines such as RSV, it is crucial that policy makers and those involved in implementation of new vaccines understand the relevance of this data, and invest in and build robust systems for capturing this data.

Access to ANC services for all pregnant women is a priority to reduce perinatal and maternal mortality. Transitioning to eight visits will likely help reduce the missed opportunities for vaccination and other life-saving interventions due to increased contacts with pregnant women. Early ANC contact is essential to screen for and prevent or treat any important morbidities as early as possible, assess risk of developing pregnancy related complications and plan for relevant interventions as well as establish gestational age more accurately, while more frequent visits in the latter part of the pregnancy may assist with immunization coverage and reducing stillbirths [23], [24]. Populations with suboptimal access early in pregnancy (some due to geographical remoteness or women not recognising the need for care early in pregnancy) provide additional challenges to antenatal care service delivery. Countries struggling to raise TT2 + coverage often have dispersed populations. In these settings outreach services are extremely important but often under-resourced.

Whilst building robust systems to capture data on timing and number of antenatal contacts, consideration should be given to a coordinated approach across information systems. In-particular, improvements in tracking, reporting, linkage of mother and child records, and surveillance systems for maternal and neonatal morbidity and mortality would enable planning for the introduction of additional maternal vaccines in the future.

Uncertainty about assessing quality of antenatal care notwithstanding, the issue of out-of-pocket expenses for women is relevant to the quality of antenatal care and attendance for the recommended minimum number of antenatal visits. The presence of user fees was significantly associated with lower PAB coverage. This is likely because user fees may be a disincentive for women to access healthcare services. If increased utilisation of ANC services is required to maximise the opportunities for women to receive maternal vaccines, then any disincentives such as out of pocket expenses need to be addressed. In addition, hidden costs for women such as transport to and from the facilities and long waiting times may further discourage utilization of antenatal care services. In the MIACSA project, out of pocket expenses did not necessarily apply to vaccination but to other antenatal care services such as pathology or ultrasound as well as cost for transportation. In all but one of the 95 countries surveyed online, maternal immunization in the public system incurred no cost to the woman. However, the free cost of the vaccine was undermined in instances when the woman accessed the vaccine through ANC services for which there were out-of-pocket expenses.

A well-functioning disease surveillance system for both maternal and neonatal mortality and morbidity is crucial to inform the need and performance of any existing or future vaccine programme. Based on the findings from the MIACSA project there is significant variability, depending on the setting, on the range of diseases covered by surveillance and often a complete absence of a surveillance system for outcomes such as stillbirth. These gaps and inconsistencies identified by the MIACSA project highlight the need to strengthen capacity for both maternal and neonatal disease surveillance in parallel with strengthening antenatal care service delivery. These systems are essential to the planning, implementation and the evaluation of any future public health initiative such as a new maternal immunization programme.

The study methodology has several limitations. The different data sources used in the MIACSA project meant that inconsistencies between the different databases were observed for some quantitative and policy-related indicators. This may have been due to collection of information at different time-points, inconsistent definitions across sources, or different sampling methodologies. Besides different sources of data, inaccurate reporting cannot be fully excluded despite efforts made by the countries to validate the information provided to the study team. Another limitation was the relatively small number of countries visited, and of health facilities by country. Health facilities visited were not randomly selected but were assigned by the Ministries of Health. They are not therefore necessarily representative of the entire country but rather reflect a potentially higher-performing part of the health system within a particular country.

## 5 Conclusion

The MIACSA project has identified aspects of maternal tetanus immunization that need to be strengthened and gaps that need to be addressed to inform the introduction of additional maternal vaccines particularly into antenatal care services. Specific recommendations arising from the MIACSA project include;

• Increasing the number of countries adopting the current WHO recommendation for eight antenatal contacts thereby increasing opportunities for vaccination during pregnancy
• Introducing record keeping systems that are able to identify the number of visits women attend during pregnancy
• Introducing record keeping systems that are able to identify the timing of visits during pregnancy (not just total number)
• Reducing any associated costs for ANC and supporting user fee exemptions for all vulnerable women
• Introducing maternal and neonatal disease surveillance systems (including for stillbirth) consistently across all LMICs

It is recognised that these recommendations require a substantial financial and political commitment depending on the individual country context. In addition, these recommendations may be prioritised differently according to existing country context, record keeping systems, surveillance systems and models of antenatal care. However, it is envisaged that policy makers and program managers for immunization and antenatal care programs are encouraged to consider these and contemplate how they can be addressed in their local setting.

It is hoped that with implementation of these recommendations a stronger ANC system will prevail leading to improved utilisation of ANC services. This in turn provides greater opportunities to vaccinate pregnant women with the currently recommended maternal vaccines and a robust platform for the introduction of new maternal vaccines in the future.

## Declaration of Competing Interest

The authors declare that they have no known competing financial interests or personal relationships that could have appeared to influence the work reported in this paper.
